# Hoping to Adhere? Examining the Relationship Between Hope and Pre-exposure Prophylaxis Willingness, Adherence, and Persistence Among Young Women in South Africa and Zimbabwe (HPTN 082)

**DOI:** 10.1007/s10461-024-04536-3

**Published:** 2024-10-24

**Authors:** Margaret W. Gichane, Jennifer Velloza, Sybil Hosek, Geetha Beauchamp, Peter Anderson, Sinead Delany-Moretlwe, Connie Celum, and the HPTN 082 Study Team

**Affiliations:** 1 Department of Obstetrics, Gynecology & Reproductive Sciences, Advancing New Standards in Reproductive Health, University of California, San Francisco, Oakland, CA USA; 2 Division of Global Health and Infectious Disease Epidemiology, University of California San Francisco, San Francisco, USA; 3 Department of Medicine, University of Illinois Chicago, Chicago, USA; 4 Fred Hutchinson Cancer Center, Seattle, USA; 5 University of Colorado Denver - Anschutz Medical Campus, Denver, USA; 6 Wits RHI, Faculty of Health Sciences, University of the Witwatersrand, Johannesburg, South Africa; 7 Departments of Global Health, Medicine, and Epidemiology, University of Washington, Seattle, USA

**Keywords:** Hope, PrEP adherence, PrEP persistence, Young women, Africa

## Abstract

Hope is a powerful psychological construct which is linked to positive health. Greater hope is associated with improved antiretroviral therapy adherence; however, less is known about the impact of hope on oral pre-exposure prophylaxis (PrEP) outcomes. HIV Prevention Trials Network 082, was an open-label PrEP study among young women (ages 16–25) in South Africa and Zimbabwe. Hope was measured at baseline and follow-up using a subset of the Hope for the Future Scale (score range 6–24) and PrEP willingness was measured using a subscale of the HIV Prevention Readiness Measure (score range 6–30). Intracellular tenofovir-diphosphate (TFV-DP) concentrations were obtained from dried blood spot samples at weeks 13, 26, and 52; high PrEP adherence was defined as TFV-DP concentrations ≥ 700 fmol/punch. Persistence was defined as TFV-DP > 16 fmol/punch at weeks 26 and 52. Linear regression and generalized estimating equations were used to assess the relationship between hope and PrEP willingness, adherence, and persistence. The median age of participants (n = 432) was 21 years (interquartile range [IQR]: 19–22). The mean hope score at baseline was 21.0 (SD = 3.4). Although hope was positively associated with PrEP willingness (β = 0.22, 95% CI 0.15, 0.37), it was not associated with high PrEP adherence (aRR = 1.00, 95% CI 0.96, 1.05), or persistence at follow-up (aRR = 1.02, 95% CI 0.99, 1.05). While cultivating hope may be an important strategy in building willingness to take oral PrEP, it may not be enough to sustain PrEP adherence or persistence.

**Supplementary Information:**

The online version contains supplementary material available at 10.1007/s10461-024-04536-3.

## Introduction

“Hope” is positive future expectancy, theorized as an individual’s agency and capacity to reach their goals within their structural context [1]. Hopefulness is linked to resilience during diagnosis, treatment, and long term prognosis of various health conditions [2, 3]. In the context of HIV, improvements in access and efficacy of antiretroviral therapy (ART) have led to growing recognition of the role of hope in the well-being of people living with HIV [4, 5]. A study of adolescents living with HIV found that hope for the future motivated their ART adherence due to aspirations to meet educational, career, and family building goals [6]. Additionally, studies of adults living with HIV have found positive associations between hope and ART adherence and engagement in HIV care [7], CD4 values [8], and improved quality of life [9].

Emerging evidence suggests that hope may also influence HIV prevention. Hope for the future is associated with self-efficacy related to higher condom use [10] and delayed sexual debut [11] among adolescent girls and young women in South Africa. In early studies of pre-exposure prophylaxis (PrEP) implementation with individuals in sero-different heterosexual relationships, couples reported that PrEP offered them a sense of hope for proximal desires of safety and normalcy, as well as longer term goals for having children [12, 13]. Hope may motivate individuals to take and adhere to PrEP as indicated by a study of South African female sex workers which found that PrEP was viewed as a source of hope [14]. However, the relationship between hope and PrEP adherence and persistence has not been studied in the priority population of East and Southern African adolescent girls and young women (AGYW), who continue to have high HIV incidence.

Studies to date indicate that African AGYW often have high levels of PrEP uptake but low adherence and experience a significant drop off in PrEP use in the first three months following initiation [15–17]. Cultivating hope among young women may help address known barriers to PrEP including pill fatigue and forgetfulness [18–20]. The purpose of this study was to examine the relationship between hope for the future and PrEP willingness, adherence, and persistence among young women in South Africa and Zimbabwe.

## Methods

### Study Design and Participants

HIV Prevention Trials Network (HPTN) 082 was an open-label PrEP demonstration study among young women in Cape Town and Johannesburg, South Africa, and Harare, Zimbabwe conducted between 2016 and 2018 [21]. To be eligible, participants had to be HIV negative, between 16 and 25 years old, sexually active and at high risk of HIV acquisition [22]. A total of 451 participants were enrolled in the study, all of whom were offered daily oral PrEP. Those who initiated PrEP were randomized in a 1:1 ratio to the control group with a standard PrEP adherence package (counselling sessions, weekly SMS reminders, monthly in-person adherence support clubs) or the enhanced adherence intervention group (the standard package plus counselling based on PrEP drug levels, called “drug-level feedback counselling”).

### Data Collection

Sociodemographic, psychosocial, and behavioral data were collected using computer-assisted self-interviewing (CASI) methods at enrolment and follow-up timepoints. Participants completed surveys providing data on social support, sexual behavior (condom use, transactional sex), intimate partner violence (IPV), and depressive symptoms. Depressive symptoms were measured using the 10-item Center for Epidemiologic Studies (CES-D). A score ≥ 10 was indicative of elevated depressive symptoms. This score has been previously used in studies of African adolescents and young adults, including in several study languages (isiZulu, IsiXhosa) [23–25]. Social support was measured with two items assessing the extent to which participants felt supported by close friends and adults. Items were adapted from prior work with young African women [26].

“Hope for the future” was measured at baseline and 39 weeks with a six-item scale which assessed anticipation for a positive future and motivation for goal achievement. Responses were on a four-point Likert scale (totally disagree, disagree, agree, totally agree). Response values were added to create a summary hope score with higher scores indicating more hope (range 6–24) [10]. The Cronbach’s alpha for the hope scale was 0.92.

PrEP willingness was measured at baseline using a subset of questions from the HIV Prevention Readiness Measure (HPRM). Responses were on a five-point Likert scale (totally disagree, disagree, “neither agree to disagree”, agree, totally agree). Response values were added to create a summary PrEP willingness score with higher scores indicating more willingness (range 6–30) [27].

PrEP adherence was measured using intracellular tenofovir-diphosphate levels (TFV-DP) in dried blood spots (DBS). TFV-DP is a measure of sustained adherence and provides a picture of the dosage and average use of PrEP in the past month. DBS samples were collected at collected at three, six, and twelve-month follow-up. PrEP adherence was treated as a binary measure. For binary analyses, we used a cutoff of TFV-DP ≥ 700 fmol/punch for high adherence. The threshold of high adherence was selected due to evidence that dosing of 700 fmol/punch was associated with 100% PrEP efficacy in men who have sex with men [28]. Persistence was measured as detectable PrEP (> 16 fmol/punch) at 26 and 52 weeks.

### Data Analysis

We analyzed data using Stata v17. We used Wilcoxon–Mann–Whitney and Kruskall Wallis tests to examine differences in hope scores by baseline characteristics. We conducted linear regression to examine the association between hope and PrEP willingness at baseline. We used generalized estimating equations with a log link, Poisson distribution, exchangeable correlation structure, and robust standard errors to estimate risk ratios for the association between baseline hope and high adherence and persistence at follow-up. Any baseline factors associated with hope (based on p < 0.10) were included in the multivariable models. Multivariable models also included randomization arm and site as covariates to adjust for differences in the demographic characteristics of participants from each city and any potential impacts of receipt of the enhanced adherence support intervention. Additionally, we ran sensitivity analyses assuming missing data indicated not achieving high adherence and non-detectable PrEP levels at follow-up.

### Ethical Review

The study protocol was approved by the ethics review committees at each study site: University of Cape Town Faculty of Health Sciences (reference number: 129/2016), University of Witwatersrand, Human Research Ethics Committee (reference number: 160304), and University of Zimbabwe Joint Research Ethics Committee (reference Number: 27/16). All participants provided written informed consent in English or their local language. Following local regulations, participants below the legal age for consent provided assent, and parent or guardian informed consent was obtained.

## Results

### Participant Characteristics

A total of 451 participants were enrolled in HPTN 082, of whom 432 completed baseline questions about hope for the future (Table 1). The median age was 21 years (interquartile range [IQR]: 19–22). Forty-one percent of participants were currently enrolled in school, and 85% had some secondary education or higher (85%). Three quarters of participants reported that they sometimes or often worried about food security in their households. The majority of participants (88%) reported having a primary sexual partner. A third of participants reported rarely or never using condoms during vaginal sex in the past month. A minority (28%) reported engaging in transactional sex in the past month. Missing hope scores were associated with having support from adults, support from friends, and having a primary sexual partner (Appendix A).

**Table 1:** Baseline characteristics by mean hope score

Characteristic	Overall (n = 432)	Mean (SD)	Statistic	p-values
Demographic				
Age^a^				
16–19	132 (31%)	20.8 (3.4)	− 1.427	0.1535
20–26	300 (69%)	21.1 (3.3)		
Enrolled in school				
Yes	175 (41%)	21.7 (3.3)	− 4.088	** < 0.001**
No	257 (59%)	20.6 (3.4)		
Education^b^				
Primary	9 (2%)	18.8 (4.1)	7.179	**0.0243**
Secondary	369 (85%)	21.0 (3.3)		
College or University	54 (13%)	21.6 (3.7)		
Ever dropped out of school				
Yes	122 (28%)	20.3 (3.5)	3.308	**0.0009**
No	310 (72%)	21.3 (3.3)		
Food security (n = 423)^b^				
Never worried	104 (25%)	21.0 (3.6)	2.584	0.2623
Sometimes worried	251 (59%)	20.9 (3.3)		
Often worried	68 (16%)	21.5 (3.1)		
Support				
Lives with parents^a^				
Yes	211 (49%)	21.0 (3.5)	0.112	0.9112
No	221 (51%)	21.1 (3.2)		
Lives with partner^a^				
Yes	95 (22%)	20.6 (2.8)	2.497	**0.0125**
No	337 (78%)	21.1 (3.5)		
Support from adults (n = 428)^b^				
Almost never supported	23 (5%)	20.9 (3.9)	0.803	0.6692
Sometimes supported	150 (35%)	21.0 (3.1)		
Very well supported	255 (60%)	21.0 (3.5)		
Support from friends (n = 426)^b^				
Almost never supported	22 (5%)	22.1 (2.3)	2.817	0.2445
Sometimes supported	223 (52%)	21.1 (3.1)		
Very well supported	181 (43%)	20.8 (3.8)		
Behavioral and psychosocial factors				
Has a primary sex partner, past 3 months (n = 422)^a^				
Yes	370 (88%)	21.2 (3.1)	− 2.057	**0.0397**
No	52 (12%)	19.7 (4.7)		
Condom use with vaginal sex, past month (n = 343)^b^				
Never	65 (20%)	21.3 (3.1)	0.647	0.9577
Rarely	52 (15%)	21.5 (3.1)		
Sometimes	118 (34%)	21.1 (3.5)		
Often	37 (11%)	21.1 (3.9)		
Always	71 (20%)	21.5 (2.6)		
Any transactional sex in the past month^a^				
Yes	122 (28%)	20.1 (3.6)	− 3.967	**0.0001**
No	310 (72%)	21.4 (3.2)		
Any emotional violence from partner, past year (n = 428)^a^				
Yes	160 (37%)	21.0 (3.3)	0.769	0.442
No	268 (63%)	21.1 (3.5)		
Any physical violence from partner, past year (n = 430)^a^				
Yes	84 (19%)	20.3 (3.9)	2.277	**0.0228**
No	346 (81%)	21.2 (3.2)		
Any sexual violence from partner, past year (n = 431)^a^				
Yes	40 (9%)	21.0 (4.2)	− 0.605	0.5452
No	391 (91%)	21.0 (3.3)		
CESD scale > 10				
Yes	184	20.7	2.124	**0.0337**
No	248	21.3		
Covariates				
Intervention Group (n = 409)^a^				
Enhanced adherence	209 (51%)	21.1 (3.5)	1.564	0.1179
Standard	200 (49%)	20.8 (3.3)		
Study Site^b^				
Cape Town	134 (31%)	20.9 (3.6)	31.962	**0.0001**
Johannesburg	156 (36%)	21.9 (3.1)		
Harare	142 (33%)	20.1 (3.2)		

^*a*^Data were not normally distributed; thus, we used nonparametric Wilcoxon–Mann–Whitney test to assess differences across groups. The z score statistic is reported.

^*b*^Data were not normally distributed; thus, we used nonparametric Kruskal–Wallis test to assess differences across groups. The chi^2^ statistic is reported.

The median hope score was 22 at baseline (IQR: 18.5, 24). Hope was consistent between baseline (M = 21.0, SD = 3.4) and 39-week follow-up (M = 21.0, SD = 2.9), t (744) = − 0.045, p = 0.96). For each hope item, over 90% of participants reported that they agreed or strongly agreed (Table 2). Mean levels of hope were higher among participants who had never dropped out of school, compared to those who had ever dropped out (M = 21.3 vs. M = 20.3, p < 0.0001), and among participants currently enrolled in school compared to those not enrolled (M = 21.7 vs. M = 20.6, p < 0.001). In terms of support, hope was higher among participants who did not live with a partner compared to those who did live with a partner (M = 21.1 vs. M = 20.6, p = 0.0125). Among psychosocial and behavioral outcomes, hope levels were lower among participants who did not have a primary sexual partner (M = 19.7 vs. M = 21.2, p = 0.0397), had engaged in transactional sex in the past month (M = 20.1 vs. M = 21.4, p < 0.0001), and among those who reported any history of physical violence (M = 20.3 vs. M = 21.2, p = 0.0228). Participants with a CESD score above 10 had lower hope levels compared to those with a score below 10 (M = 20.7 vs. M = 21.3, p = 0.0337).

**Table 2:** Distribution of hope items

Item		Totally disagree, n (%)	Disagree, n (%)	Agree, n(%)	Totally agree, n(%)
*Personal motivation to achieve goals*					
3	I trust that I will achieve the goals that I set for myself	14 (3.1)	4 (0.9)	150 (33.6)	279 (62.4)
6	I can achieve my dreams if I focus on them	12 (2.7)	8 (1.8)	147 (32.9)	280 (62.6)
*Anticipation of a positive future*					
1	I know that my life will be better in the future	12 (2.7)	2 (0.5)	165 (37.3)	264 (59.6)
4	I believe that the things I am doing now are preparing me for what I want in the future	12 (2.7)	7 (1.6)	182 (40.9)	244 (54.8)
5	I believe that I will be successful even when there are difficulties in my life now	15 (3.4)	2 (0.5)	164 (36.7)	266 (59.5)
*Influence of others on hope*					
2	The important people in my life tell me that I will have a successful life	17 (3.9)	17 (3.9)	213 (48.3)	194 (43.0)

### Association Between Hope for the Future and PrEP Willingness, Adherence, and Persistence

At baseline 418 participants reported on PrEP willingness. Overall, participants reported a high willingness to use PrEP (M = 25.9, SD = 3.9). For every one-standard deviation increase in hope, there was a 0.22 standard deviation increase in PrEP willingness (β = 0.22, p < 0.001).

Four hundred and twenty-seven participants initiated PrEP. DBS data were available for 357, 348, 333, participants at weeks 13, 26, and 52 respectively. The number of participants with high adherence (> 700fmol/punch) declined over time. At 13 weeks, 25% of participants had high adherence based on the > 700 fmol/punch threshold which decreased to 22% participants at week 26, and 9% of participants at week 52. In multivariable models, baseline hope levels were not associated with high PrEP adherence at follow-up timepoints (aRR = 1.00, 95% CI 0.96, 1.05) (Table 3). Similarly, hope was not associated with high PrEP adherence at follow-up in sensitivity analysis (RR = 0.99, 95% CI 0.94, 1.02).

**Table 3:** Longitudinal association between hope and PrEP adherence

	RR	95% CI	p-value	aRR^a^	95% CI	p-value
High adherence						
Hope	0.99	0.95, 1.04	0.73	1.00	0.96, 1.05	1.00
Persistence						
Hope	1.01	0.98, 1.04	0.418	1.02	0.99, 1.05	0.303

*RR* relative risk, *aRR* adjusted relative risk

^a^Multivariable models adjust for education, school enrollment, school dropout, living with a primary partner, transactional sex engagement, experience of physical intimate partner violence in the past year, site and study arm.

Fifty-eight percent of participants had detectable PrEP levels at 26 weeks and 32% of participants had detectable TFV-DP levels at 52 weeks. In multivariable models, baseline hope was not associated with PrEP persistence at follow-up time points (aRR = 1.02, 95% CI 0.99, 1.05) (Table 3). Similarly, hope was not associated with PrEP persistence at follow-up in sensitivity analysis (RR = 1.00, 95% CI 0.97, 1.03).

## Discussion

In a study of young women enrolled in an open label HIV prevention trial, we explored the relationship between hope for the future and PrEP outcomes. Overall, we found that participant hope scores were near the top of the scale range, and that scores remained stable between baseline and 39 weeks. Hope was modestly positively correlated with willingness to use PrEP. However, we did not find a significant association between hope and PrEP adherence or persistence. Our study adds to growing evidence exploring the role of hope in HIV prevention.

Although the positive relationship between hope and PrEP willingness was small, previous literature linking hopelessness to adolescent sexual risk behaviors [29, 30] suggests that building hope may be a promising strategy in HIV prevention interventions. A recent review found that hopelessness is amenable to change via strategies such as therapy, behavioral interventions, support groups, and individual exercises [31]. One such example is the Building Hope for the Future Intervention which was tested in a sample of middle school students [32]. The five-week group-based intervention focused aimed to help students to (1) conceptualize clear goals; (2) produce a numerous range of pathways to attainment; (3) summon the mental energy to maintain the goal pursuit; and (4) reframe seemingly insurmountable obstacles as challenges to be overcome. Program completion was associated with gains in hope, life satisfaction and self-worth up to 18 months post intervention [32]. Evidence of the association between neighborhood [33] and household [11] socioeconomic conditions and hope, also suggest that structural interventions which aim to change the conditions which restrict hope, including through providing educational and economic support, may increase hope, and in turn impact HIV prevention behaviors. These strategies warrant further research in PrEP delivery programs, including offering a choice of long-acting PrEP which may be less burdensome for women than daily pill-taking and provide them with a greater sense of autonomy.

The null relationships between hope and PrEP adherence and persistence are contrary to literature examining the relationship between hope and adherence to ART [7]. There are several possible explanations for this finding. First, adolescence is a period of rapid developmental and neurocognitive changes. During this period adolescents show a strong present bias [34] which makes them more likely to weigh the present demands and ‘costs’ of taking PrEP (e.g. potential side effects, waiting at a clinic, etc.) [35] compared to the potential long term benefit of not acquiring HIV. Second, hope may be a better determinant of adherence for conditions which are already diagnosed, compared to HIV acquisition for some adolescent populations. Hope is associated with treatment adherence in studies on adolescent cancer [36], asthma [37] and type 1 diabetes [38]. Third, hope may have a differential impact on preventative behaviors. A study among young women in Durban, South Africa found positive relationships between hope and modern contraceptive use, but null associations between HIV risk and protective behaviors including transactional sex and condom use [39]. Though adolescent girls and young women may seek to both prevent unintended pregnancy and HIV, factors including stigma of being perceived to be living with HIV [20], perceived risk of HIV acquisition versus pregnancy, and burden of use (e.g. daily oral PrEP vs. contraceptive injection every three months) may increase their motivation to use contraception in comparison to oral PrEP. Future research should explore the association between hope and PrEP adherence for diverse adolescent populations and with introduction of highly efficacious, and less adherence dependent, longer-acting PrEP formulations.

There was limited variability in hope scores. With nearly all participants reporting high hope scores, we were restricted in our ability to understand the full spectrum of hope scores and the relationship between PrEP outcomes. This finding is similar to previous attempts to measure hope in the context of HIV prevention among women in Eastern and Southern Africa [10, 39, 40]. The Hope for the Future scale used in this study was first developed and validated with young women within the context of HPTN 068, a randomized trial which assessed the impact of conditional cash incentives on the incidence of HIV, HSV-2, and sexual risk behaviors in Mpumalanga province, South Africa. In HPTN 068 over 75% of young women enrolled in the trial reported high levels of hope [10]. Similarly, hope was assessed among women using the Snyder Hope Scale in trials for MAISHA [40], a microfinance and intimate partner violence (IPV) prevention intervention implemented in Tanzania, and Stepping Stones and Creating Futures [39], an IPV and livelihoods strengthening intervention implemented in South Africa. In all three studies, most participants had hope scores that were at the midpoint or higher, with limited variation in scores. While intervention trials may recruit participants with higher hope compared to general populations [41], there is a need for measures which capture a fuller range of hope.

These findings must be interpreted in light of a few limitations. We used a baseline measure of hope to assess adherence and persistence at 3-, 6-, and 12-month follow-up. While we found that hope was stable between baseline and 39 weeks (~ 10 months), it is possible that we did not fully capture variations in hope that were more proximal to DBS measurement. The large drop offs in PrEP adherence and persistence over time may have also reduced our ability to detect the effect of hope. Further, HPTN 082 trial was not designed with a primary focus on the impact of hope on PrEP outcomes thus we may be missing data on other key confounders. HPTN 082 was implemented from 2016 to 2018 during the early roll out of oral PrEP in South Africa and Zimbabwe. Limited awareness about PrEP among the general public may have made it difficult for young women to store their bottle of pills and to adhere or persist to daily pill-taking for HIV prevention with a drug that many associated with HIV treatment. Finally, this analysis was done in the context of a clinical trial providing a large package of supportive PrEP adherence counseling interventions—results on the association between hope and PrEP use may not be generalizable to young women outside of a PrEP trial setting.

## Conclusion

Our study provides novel evidence of the relationship between hope and PrEP willingness, adherence, and persistence in a sample of young women in high HIV burden Southern African settings. Additional research to understand the relationship of hope to HIV prevention and integrating interventions which support and sustain hope among young women may be a promising strategy to explore in HIV prevention interventions.

## Supplementary Information

Below is the link to the electronic supplementary material.

Supplementary file1 (DOCX 21 kb)

## References

[1] Snyder CR, Harris C, Anderson JR, Holleran SA, Irving LM, Sigmon ST, et al. The will and the ways: development and validation of an individual-differences measure of hope. J Pers Soc Psychol. 1991;60(4):570. 2037968 10.1037//0022-3514.60.4.570

[2] Rasmussen HN, O’Byrne KK, Vandament M, Cole BP. Hope and physical health. In: Gallagher MWL, Shane J, editors. The Oxford handbook of hope. New York: Oxford University Press; 2018. p. 159–68.

[3] Snyder CR, Irving LM, Anderson JR. Hope and health: measuring the will and the ways. In: Snyder CR, Forsyth DR, editors. Handbook of social and clinical psychology: the health perspective. Elmsford: Pergamo; 1991. p. 285–305.

[4] Barnett T, Seeley J, Levin J, Katongole J. Hope: a new approach to understanding structural factors in HIV acquisition. Glob Public Health. 2015;10(4):417–37. 25648679 10.1080/17441692.2015.1007154

[5] Bernays S, Rhodes T, Barnett T. Hope: a new way to look at the HIV epidemic. AIDS. 2007;21:S5–11. 18090269 10.1097/01.aids.0000298097.64237.4b

[6] Bakeera-Kitaka S, Nabukeera-Barungi N, Nöstlinger C, Addy K, Colebunders R. Sexual risk reduction needs of adolescents living with HIV in a clinical care setting. AIDS Care. 2008;20(4):426–33. 18449819 10.1080/09540120701867099

[7] Siril H, Smith Fawzi MC, Todd J, Somba M, Kaale A, Minja A, et al. The value of hope: development and validation of a contextual measure of hope among people living with HIV in urban Tanzania a mixed methods exploratory sequential study. BMC Psychology. 2020;8(1):5. 31996246 10.1186/s40359-020-0376-y PMC6988347

[8] Scioli A, MacNeil S, Partridge V, Tinker E, Hawkins E. Hope, HIV and health: a prospective study. AIDS Care. 2012;24(2):149–56. 21780957 10.1080/09540121.2011.597943

[9] Yadav S. Perceived social support, hope, and quality of life of persons living with HIV/AIDS: a case study from Nepal. Qual Life Res. 2010;19(2):157–66. 20047029 10.1007/s11136-009-9574-z

[10] Abler L, Hill L, Maman S, DeVellis R, Twine R, Kahn K, et al. Hope matters: developing and validating a measure of future expectations among young women in a high HIV prevalence setting in rural South Africa (HPTN 068). AIDS Behav. 2017;21(7):2156–66. 27544516 10.1007/s10461-016-1523-6 PMC5626443

[11] Hill LM, Abler L, Maman S, Twine R, Kahn K, MacPhail C, et al. Hope, the household environment, and sexual risk behaviors among young women in rural South Africa (HPTN 068). AIDS Behav. 2018;22(6):1908–18. 29076034 10.1007/s10461-017-1945-9 PMC5920793

[12] Idoko J, Folayan MO, Dadem NY, Kolawole GO, Anenih J, Alhassan E. “Why should I take drugs for your infection?”: outcomes of formative research on the use of HIV pre-exposure prophylaxis in Nigeria. BMC Public Health. 2015;15(1):349. 25881087 10.1186/s12889-015-1690-9 PMC4404045

[13] Ware NC, Wyatt MA, Haberer JE, Baeten JM, Kintu A, Psaros C, et al. What’s love got to do with it? Explaining adherence to oral antiretroviral pre-exposure prophylaxis for HIV-serodiscordant couples. J Acquir Immune Defic Syndr. 2012;59(5):463–8. 22267018 10.1097/QAI.0b013e31824a060b PMC3826169

[14] Makhakhe NF, MeyerWeitz A, Sliep Y. Motivating factors associated with oral pre-exposure prophylaxis use among female sex workers in South Africa. J Health Psychol. 2022;27(12):2820–33. 34991407 10.1177/13591053211072674

[15] Celum CL, Gill K, Morton JF, Stein G, Myers L, Thomas KK, et al. Incentives conditioned on tenofovir levels to support PrEP adherence among young South African women: a randomized trial. J Int AIDS Soc. 2020;23(11): e25636. 33247553 10.1002/jia2.25636 PMC7695999

[16] Mansoor LE, Lewis L, Naicker CL, Harkoo I, Dawood H, Naidoo K, et al. Prospective study of oral pre-exposure prophylaxis initiation and adherence among young women in KwaZulu-Natal, South Africa. J Int AIDS Soc. 2022;25(7): e25957. 35785472 10.1002/jia2.25957 PMC9251857

[17] Elzette R-J, Linda-Gail B, Elizabeth B, Sinead D-M, Victor O, Danielle T, et al. Early persistence of HIV Pre-exposure prophylaxis (PrEP) in african adolescent girls and young women (AGYW) from Kenya and South Africa. AIDS Res Hum Retroviruses. 2018;34(S1):1–407.29161879

[18] Bjertrup PJ, Mmema N, Dlamini V, Ciglenecki I, Mpala Q, Matse S, et al. PrEP reminds me that I am the one to take responsibility of my life: a qualitative study exploring experiences of and attitudes towards pre-exposure prophylaxis use by women in Eswatini. BMC Public Health. 2021;21(1):727. 33853575 10.1186/s12889-021-10766-0 PMC8048211

[19] Cassidy T, Ntuli N, Kilani C, Malabi N, Rorwana B, Mutseyekwa T, et al. Delivering PrEP to Young Women in a Low-Income Setting in South Africa: Lessons for Providing Both Convenience and Support. AIDS and Behavior. 2021. 10.1007/s10461-021-03366-x PMC8786762 34259963

[20] Velloza J, Khoza N, Scorgie F, Chitukuta M, Mutero P, Mutiti K, et al. The influence of HIV-related stigma on PrEP disclosure and adherence among adolescent girls and young women in HPTN 082: a qualitative study. J Int AIDS Soc. 2020;23(3): e25463. 32144874 10.1002/jia2.25463 PMC7060297

[21] Celum C, Hosek S, Tsholwana M, Kassim S, Mukaka S, Dye BJ, et al. PrEP uptake, persistence, adherence, and effect of retrospective drug level feedback on PrEP adherence among young women in southern Africa: results from HPTN 082, a randomized controlled trial. PLoS Med. 2021;18(6): e1003670. 34143779 10.1371/journal.pmed.1003670 PMC8253429

[22] Balkus JE, Brown E, Palanee T, Nair G, Gafoor Z, Zhang J, et al. An empiric HIV risk scoring tool to predict HIV-1 acquisition in African women. JAIDS J Acquir Immune Defic Syndr. 2016;72(3):333–43. 26918545 10.1097/QAI.0000000000000974 PMC4911322

[23] Baron EC, Davies T, Lund C. Validation of the 10-item centre for epidemiological studies depression scale (CES-D-10) in Zulu, Xhosa and Afrikaans populations in South Africa. BMC Psychiatry. 2017;17(1):6. 28068955 10.1186/s12888-016-1178-x PMC5223549

[24] Kilburn K, Prencipe L, Hjelm L, Peterman A, Handa S, Palermo T. Examination of performance of the center for epidemiologic studies depression scale short form 10 among African youth in poor, rural households. BMC Psychiatry. 2018;18(1):201. 29914413 10.1186/s12888-018-1774-z PMC6006708

[25] Oppong Asante K, Andoh-Arthur J. Prevalence and determinants of depressive symptoms among university students in Ghana. J Affect Disord. 2015;171:161–6. 25305431 10.1016/j.jad.2014.09.025

[26] Cowan FM, Davey C, Fearon E, Mushati P, Dirawo J, Chabata S, et al. Targeted combination prevention to support female sex workers in Zimbabwe accessing and adhering to antiretrovirals for treatment and prevention of HIV (SAPPH-IRe): a cluster-randomised trial. Lancet HIV. 2018;5(8):e417–26. 30030134 10.1016/S2352-3018(18)30111-5

[27] Beauchamp G, Hosek S, Donnell DJ, Chan KCG, Flaherty BP, Anderson PL, et al. Development of a tool to assess HIV prevention readiness of adolescent girls and young women in HPTN 082 study. PLoS ONE. 2023;18(2): e0281728. 36827440 10.1371/journal.pone.0281728 PMC9956790

[28] Grant RM, Anderson PL, McMahan V, Liu A, Amico KR, Mehrotra M, et al. Uptake of pre-exposure prophylaxis, sexual practices, and HIV incidence in men and transgender women who have sex with men: a cohort study. Lancet Infect Dis. 2014;14(9):820–9. 25065857 10.1016/S1473-3099(14)70847-3 PMC6107918

[29] James S, Reddy SP, Ellahebokus A, Sewpaul R, Naidoo P. The association between adolescent risk behaviours and feelings of sadness or hopelessness: a cross-sectional survey of South African secondary school learners. Psychol Health Med. 2017;22(7):778–89. 28290218 10.1080/13548506.2017.1300669

[30] Kagan S, Deardorff J, McCright J, Lightfoot M, Lahiff M, Lippman SA. Hopelessness and sexual risk behavior among adolescent African American males in a low-income urban community. Am J Mens Health. 2012;6(5):395–9. 22406766 10.1177/1557988312439407 PMC4475028

[31] Marchetti I, Alloy LB, Koster EHW. Breaking the vise of hopelessness: targeting its components, antecedents, and context. Int J Cogn Ther. 2023;16(3):285–319. 39131585 10.1007/s41811-023-00165-1 PMC11314313

[32] Marques SC, Lopez SJ, Pais-Ribeiro JL. “Building hope for the future”: a program to foster strengths in middle-school students. J Happiness Stud. 2011;12(1):139–52.

[33] Morselli D. Contextual determinants of hopelessness: investigating socioeconomic factors and emotional climates. Soc Indic Res. 2017;133(1):373–93.

[34] Chesson HW, Leichliter JS, Zimet GD, Rosenthal SL, Bernstein DI, Fife KH. Discount rates and risky sexual behaviors among teenagers and young adults. J Risk Uncertain. 2006;32(3):217–30.

[35] Roy Paladhi U, Katz DA, Farquhar C, Thirumurthy H. Using behavioral economics to support PrEP adherence for HIV prevention. Curr HIV/AIDS Rep. 2022;19(5):409–14. 36044119 10.1007/s11904-022-00624-y PMC9428871

[36] Maikranz JM, Steele RG, Dreyer ML, Stratman AC, Bovaird JA. The relationship of hope and illness-related uncertainty to emotional adjustment and adherence among pediatric renal and liver transplant recipients. J Pediatr Psychol. 2006;32(5):571–81. 17145733 10.1093/jpepsy/jsl046

[37] Berg CJ, Rapoff MA, Snyder CR, Belmont JM. The relationship of children’s hope to pediatric asthma treatment adherence. J Posit Psychol. 2007;2(3):176–84.

[38] Van Allen J, Steele RG, Nelson MB, Peugh J, Egan A, Clements M, et al. A longitudinal examination of hope and optimism and their role in type 1 diabetes in youths. J Pediatr Psychol. 2015;41(7):741–9. 26628250 10.1093/jpepsy/jsv113 PMC4945775

[39] Gibbs A, Desmond C, Barnett T, Shahmanesh M, Seeley J. Is hope associated with HIV-acquisition risk and intimate partner violence amongst young women and men? A cross-sectional study in urban informal settlements in South Africa. AIDS Care. 2023;35(6):833–40. 36435964 10.1080/09540121.2022.2143470

[40] Hansen CH, Lees S, Kapiga S, Seeley J, Barnett T. Measuring hope amongst Tanzanian women who participate in microfinance: an evaluation of the Snyder hope scale. Glob Public Health. 2020;15(3):402–13. 31671282 10.1080/17441692.2019.1682027

[41] Boyce G, Harris G. Hope the beloved country: hope levels in the new South Africa. Soc Indic Res. 2013;113(1):583–97.

