# Supplementary material for: Hoping to Adhere? Examining the Relationship Between Hope and Pre-exposure Prophylaxis Willingness, Adherence, and Persistence Among Young Women in South Africa and Zimbabwe (HPTN 082)
Source: AIDS Behav. 2024 Oct 24;29(2):527–34. doi: 10.1007/s10461-024-04536-3 (PMC11814051; doi:10.1007/s10461-024-04536-3)
Supplement: Supplementary file 1 — Supplementary file1 (DOCX 21 kb) [file 10461_2024_4536_MOESM1_ESM.docx]

| **Table 1.** Characteristic of participants with missing data | | | | | |  |
| --- | --- | --- | --- | --- | --- | --- |
|  | Frequency (%) | | | | |  |
| **Characteristic** | Participants with complete data (n= 432) | | Participants with missing data (n=19) | | | p-value |
| **Demographic** |  | |  | | |  |
| *Age^a^* |  | |  | | |  |
| 16-19 | 132 (31%) | | 6 (31.6) | | | 0.925 |
| 20-26 | 300 (69%) | | 13 (68.4) | | |  |
| *Enrolled in school* |  | |  | | |  |
| Yes | 175 (41%) | | 4 (21.0) | | | 0.090 |
| No | 257 (59%) | | 15 (79.0) | | |  |
| *Education^b^* |  | |  | | |  |
| Primary | 9 (2%) | | 0 (0) | | | 0.200 |
| Secondary | 369 (85%) | | 19 (100) | | |  |
| College or University | 54 (13%) | | 0 (0) | | |  |
| *Ever dropped out of school* |  | |  | | |  |
| Yes | 122 (28%) | | 6 (31.6) | | | 0.752 |
| No | 310 (72%) | | 13 (68.4) | | |  |
| *Food security (n=438)^b^* |  | |  | | |  |
| Never worried | 104 (25%) | | 2 (13.3) | | | 0.180 |
| Sometimes worried | 251 (59%) | | 8 (53.4) | | |  |
| Often worried | 68 (16%) | | 5 (33.3) | | |  |
| **Support** | | |  | | |  |
| *Lives with parents^a^ (n=449)* | | |  | | |  |
| Yes | 211 (49%) | | 8 (47.1) | | | 0.885 |
| No | 221 (51%) | | 9 (52.9) | | |  |
| *Lives with partner (n=449)  ^a^* | | |  | | |  |
| Yes | 95 (22%) | | 3 (17.7) | | | 0.671 |
| No | 337 (78%) | | 14 (82.4) | | |  |
| *Support from adults* (n=442) *^b^* | | |  | | |  |
| Almost never supported | 23 (5%) | | 2 (14.3) | | | **0.014** |
| Sometimes supported | 150 (35%) | | 9 (64.3) | | |  |
| Very well supported | 255 (60%) | | 4 (21.4) | | |  |
| *Support* *from friends (n=426) ^b^* | |  | | |  |  |
| Almost never supported | 22 (5%) | | 4 (28.6) | | | **0.001** |
| Sometimes supported | 223 (52%) | | 4 (28.6) | | |  |
| Very well supported | 181 (43%) | | 6 (42.8) | | |  |
| **Behavioral and Psychosocial Factors** | | |  | | |  |
| *Has a primary sex partner, past 3 months (n=437) ^a^* | | |  | | |  |
| Yes | 370 (88%) | | 9 (60.0) | | | **0.002** |
| No | 52 (12%) | | 6 (40.0) | | |  |
| *Condom use with vaginal sex, past month (n = 329)^b^* | | |  | | |  |
| Never | 65 (20%) | | 4 (57.1) | | | 0.035 |
| Rarely | 52 (15%) | | 0 (0) | | |  |
| Sometimes | 118 (34%) | | 1 (14.3) | | |  |
| Often | 37 (11%) | | 2 (28.6) | | |  |
| Always | 71 (20%) | | 0 (0) | | |  |
| *Any transactional sex in the past month^a^* | | |  | | |  |
| Yes | 122 (28%) | | 6 (35.3) | | | 0.527 |
| No | 310 (72%) | | 11 (64.7) | | |  |
| *Any emotional violence from partner, past year (n=428) ^a^* | | |  | | |  |
| Yes | 160 (37%) | | 7 (46.7) | | | 0.466 |
| No | 268 (63%) | | 8 (53.3) | | |  |
| *Any physical violence from partner, past year (n=430) ^a^* | | |  | | |  |
| Yes | 84 (19%) | | 6 (37.5) | | | 0.079 |
| No | 346 (81%) | | 10 (62.5) | | |  |
| *Any sexual violence from partner, past year (n=431) ^a^* | |  | |  | |  |
| Yes | 40 (9%) | | 1 (6.3) | | | 0.680 |
| No | 391 (91%) | | 15 (93.7) | | |  |
| CESD scale >10 |  | |  | | |  |
| Yes | 184 (42.6) | | 12 (63.2) | | | 0.077 |
| No | 248 (57.4) | | 7 (36.8) | | |  |
| **Covariates** | | |  | | |  |
| *Intervention Group (n=409) ^a^* | | |  | | |  |
| Enhanced adherence | 209 (51%) | | 6 (33.3) | | | 0.140 |
| Standard | 200 (49%) | | 12 (66.7) | | |  |
| *Study Site ^b^* |  | |  | | |  |
| Cape Town | 134 (31%) | | 7 (36.8) | | | 0.856 |
| Johannesburg | 156 (36%) | | 6 (31.6) | | |  |
| Harare | 142 (33%) | | 6 (31.6) | | |  |
